# A mathematical model of serotype replacement in pneumococcal carriage following vaccination

**DOI:** 10.1098/rsif.2013.0786

**Published:** 2013-12-06

**Authors:** Christian Bottomley, Anna Roca, Philip C. Hill, Brian Greenwood, Valerie Isham

**Affiliations:** 1 MRC Tropical Epidemiology Group, LSHTM, Keppel St., London WC1E 7HT, UK; 2 Faculty of Infectious and Tropical Diseases, LSHTM, Keppel St., London WC1E 7HT, UK; 3 Medical Research Council Unit, PO Box 273, Banjul, The Gambia; 4 Centre for International Health, School of Medicine, University of Ontago, PO Box 913, Dunedin 9054, New Zealand; 5 Department of Statistical Science, University College London, Gower Street, London WC1E 6BT, UK

**Keywords:** serotype replacement, bacterial carriage, mathematical model, pneumococcal vaccine

## Abstract

A number of childhood vaccination programmes have recently introduced vaccination against *Streptococcus pneumoniae*, the pneumococcus, a major cause of pneumonia and meningitis. The pneumococcal conjugate vaccines (PCVs) that are currently in use only protect against some serotypes of the bacterium, and there is now strong evidence that those serotypes not included in the vaccine increase in prevalence among most vaccinated populations. We present a mathematical model for the dynamics of nasopharyngeal carriage of *S. pneumoniae* that allows for carriage with multiple serotypes. The model is used to predict the prevalence of vaccine type (VT) and non-VT (NVT) serotypes following the introduction of PCV. Parameter estimates for the model are obtained by maximum likelihood using pre-vaccination data from The Gambia. The model predicts that low (1, 6A and 9V) and medium (4, 5, 7F, 14, 18C, 19A and 19F) prevalence serotypes can be eliminated through vaccination, but that the overall prevalence of carriage will be reduced only slightly because of an increase in the prevalence of NVT serotypes. Serotype replacement will be sequential, with high and medium prevalence NVT serotypes dominating initially, followed by an increase of serotypes of low prevalence. We examine the impact of a hypothetical vaccine that provides partial protection against all serotypes, and find that this reduces overall carriage, but is unable to eliminate low or medium prevalence serotypes.

## 1. Background

*Streptococcus pneumoniae*, the pneumococcus, is a common cause of pneumonia, septicaemia and meningitis. It is also found frequently in the upper respiratory tract in the absence of any symptoms, a phenomenon known as nasopharyngeal carriage. The bacterium has been subclassified into 94 different serotypes on the basis of the structure of its capsular polysaccharide. Each capsular polysaccharide induces type-specific antibodies [1].

A seven-valent pneumococcal conjugate vaccine (PCV7) was introduced in The Gambia as part of the national Expanded Programme of Immunization in 2009 and was replaced by a 13-valent vaccine (PCV13) in 2011. These vaccines protect against disease caused by pneumococci of serotypes represented in the vaccine (serotypes 1, 3, 4, 5, 6A, 6B, 7F, 9V, 14, 18C, 19A, 19F and 23F in the case of PCV13), but not against other serotypes.

Results from a pneumococcal vaccine trial conducted in The Gambia, West Africa, showed that a reduction in the prevalence of nasopharyngeal carriage of vaccine type (VT) serotypes following vaccination was accompanied by an increase in non-VT (NVT) serotypes [2]. Similarly, invasive disease associated with NVT serotypes tends to increase after the introduction of PCVs. In the USA, for example, surveillance data from the CDC showed that the incidence of invasive disease in children under 2 years caused by non-vaccine serotypes increased from 13 to 19 cases per 100 000/year 4 years after the introduction of PCV7 [3]. The evidence for serotype replacement in both carriage and invasive disease following vaccination has been reviewed by Whitney & Moore [4], and recently by Weinberger *et al*. [5].

In this study, we use a mathematical model to predict the extent of the serotype replacement in carriage following the introduction of pneumococcal vaccination in The Gambia. Parameter estimates for the model are obtained from data collected before the introduction of PCV in The Gambia [6]. The model will be validated in due course, when post PCV surveys of carriage have been conducted.

## 2. Model

We model the dynamics of *S. pneumoniae* serotypes deterministically. Specifically, we assume a model of superinfection along the lines of Nowak & May [7] and Tanaka & Feldman [8], in which individuals are infected with at most one serotype at a time (see appendix for a model in which this assumption is relaxed). Serotype-specific immunity is incorporated into the model by allowing a proportion of infected individuals to develop lifelong immunity to the serotype following infection.

In a model with *n* serotypes, we must keep track of 2*^n^* immune states because an individual can be either immune or susceptible to each of the serotypes. The model becomes computationally intractable even for moderate size *n—*e.g. for *n* = 30, there are over 1 billion states. To simplify the model, we group serotypes into classes (*i* = 1,…,*m*); within a particular class, all *n_i_* serotypes are acquired and cleared at the same rate and are of the same type (i.e. VT or NVT). As all serotypes within a class are equivalent, an individual's immune state can be followed by tracking the total number of serotypes within each class to which the individual has immunity. This simplification can substantially reduce the number of possible immune states. For example, with *m* = 6 classes and five serotypes in each class, there are six states for each class (immunity to 0, 1, 2, 3, 4 or 5 serotypes) and a total of 6^6^ = 46 656 possible immune states.

To this end, we denote an immune state by *h* = (*h*_1_, *h*_2_,…,*h_m_*), where *h_i_* (0 ≤ *h_i_* ≤ *n_i_*) is the number of serotypes in class *i* to which immunity has been acquired. The following differential equations describe the dynamics of *x_h_* and 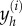, which represent, respectively, the proportions of non-carriers and carriers of a serotype in class *i* with immune state *h* among the total population. We use the notation *h_i−_* = (*h*_1_,…,*h_i_* − 1,…,*h_m_*) to represent a state where there is immunity to one fewer serotype in class *i* than there is for state *h*. We also define 
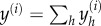
 to be the proportion of carriers of serotypes in class *i* regardless of immune state.
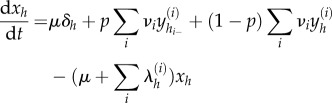
and
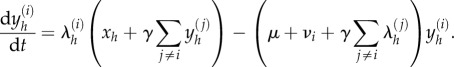


In these equations, *p* is the proportion who become immune after carriage (this is assumed to be the same for serotypes in all classes), *ν*_*i*_ are *per capita* rates of clearance, 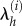 is the force of infection owing to serotypes in class *i* among non-carriers in immune state *h* and *γ* is the reduction in the rate of acquisition among carriers, relative to non-carriers. Individuals are born at a *per capita* rate *μ*, and die at the same rate, irrespective of their carriage status or immune state. The births are divided among the immune states, and the proportion born into immune state *h* is *δ*_*h*_. In the absence of vaccination, individuals are born fully susceptible to all serotypes (i.e. we neglect any maternal immunity) so that *δ*_*h*_ = 1 when *h* = (0,…,0) and zero otherwise. After vaccine introduction, we assume that a fraction, *f*, of the vaccinated children have immunity to VT serotypes at birth, while the remainder are born susceptible to all serotypes.

The *per capita* rate at which non-carriers in immune state *h* acquire serotypes of class *i* (i.e. the force of infection due to class *i* serotypes among non-carriers in state *h*) is 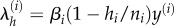, where *β*_*i*_ is the transmission parameter (*per capita* contact rate), and (1 − *h_i_*/*n_i_*) represents the proportion of serotypes in class *i* to which non-carriers are susceptible.

We have described the model in terms of the proportion of the population with immune state *h* who are carriers of a class *i* serotype. Because all serotypes in a prevalence class have the same contact and clearance rates, the proportion of the population with immune state *h* that carry a *particular* class *i* serotype is 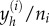, and across all immune states the proportion of carriers is 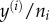. Furthermore, the model that has been described is equivalent to modelling the dynamics of all serotypes explicitly, in a model with *n* = ∑*n_i_* serotypes and *n* classes, provided that the parameters and initial conditions for the serotypes are the same in the two models. For example, a model with two serotypes and two classes is equivalent to a model with two serotypes in a single class, if in the model with two classes, both serotypes have the same contact and clearance rates, and initial prevalence. The advantage of formulating the model as above is that it substantially reduces the amount of computation.

### 2.1. Fitting the model

We parametrized the model using data collected as part of a baseline study for a community vaccine trial in the Sibanor region of The Gambia. In this study, nasopharyngeal swabs were collected from 170 subjects, of all ages, every two weeks for 50 weeks. A full description of the study is given in Hill *et al.* [6].

The model was fitted to data on the number of transitions between carriage states, where the carriage state is defined as either the absence of carriage or the presence of a particular serotype. Samples were excluded from the analysis whenever more than one serotype was isolated (the percentages of swabs with two and three serotypes were 2.95% and 0.04%, respectively).

Both VT and NVT serotypes were grouped according to their prevalence (low <0.5%, medium 0.5–2% or high >2%) in pre-vaccination data from The Gambia, which is similar to that found in other parts of Africa [9,10]. Serotype prevalence is a function of contact (*β*) and clearance (*ν*) rates, both of which are themselves positively correlated [10]; hence we expect the rates of contact and clearance to be similar among serotypes of similar prevalence.

We used the rates of transition implied by the model at equilibrium to define a likelihood in terms of probabilities of transition between carriage states in consecutive nasopharyngeal swabs. Parameter estimates were obtained by maximizing the likelihood using a Nelder–Mead algorithm and Wald-type confidence intervals are presented. The methodology used to fit the model is described in more detail in the appendix.

## 3. Results

### 3.1. Model projections

**Figure 1.**
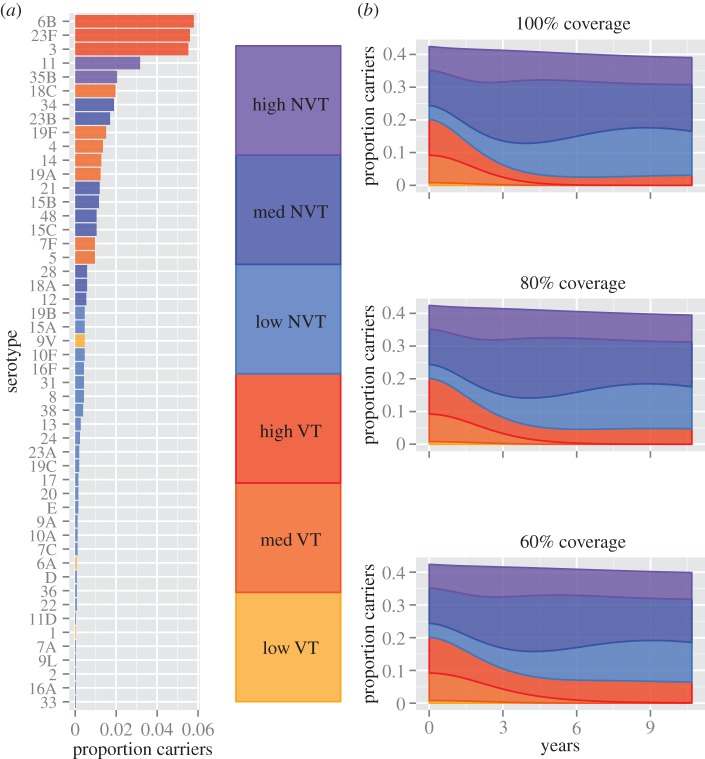
The prevalence of pneumococcal serotypes before vaccination (*a*), and the prevalence of carriage after the introduction of vaccination at three different levels of coverage (*b*).

The model predicts that the prevalence of carriage of VT serotypes will decline significantly from 20.1% before vaccination to 3.0% 10 years after vaccine introduction assuming full vaccine coverage, and 4.8% and 6.5% for 80% and 60% coverage, respectively (figure 1); low (1, 6A and 9V) and medium prevalence VT serotypes (4, 5, 7F, 14, 19A, 18C and 19F) will be driven to extinction so that only high prevalence VT serotypes (3, 6B and 23F) will continue to circulate in the population. The decline in VT serotypes will be countered by an increase in NVT serotypes from 22.3% to 36.1%, 34.8% and 33.5%, assuming 100%, 80% and 60% coverage, respectively, so that the net effect over 10 years is a small reduction in overall carriage—e.g. from 42.4 to 39.1% for full vaccine coverage. Serotype replacement will initially be dominated by serotypes from the high and medium prevalence classes, but will be followed by an expansion of low prevalence serotypes. During the first 2 years after vaccine introduction, assuming full coverage, low prevalence NVT serotypes will each increase 0.05% per year, whereas high prevalence NVT will increase by 0.36% per year. By contrast, in the following 3 years, low prevalence NVT serotypes will increase by 0.10% per year but high prevalence serotypes will decrease by 0.25% per year (see also figure 1).

### 3.2. Model fit

Parameter estimates are given in table 1. The model fits the Gambian pre-vaccination data well. The prevalence of carriage predicted by the model (VT and NVT combined) was similar to that observed: the predicted prevalence for low, medium and high prevalence serotypes was 0.056 (observed 0.058), 0.187 (observed 0.190) and 0.181 (observed 0.221), respectively, where the observed prevalence corresponds to the average prevalence from swabs collected during the study (N.B. this is less than the proportion infected at anytime in the study—85% adults, 97% children—reported in [6]). The predicted probability of transition from carriage of one serotype to another was also generally close to the observed probability (table 2).

**Table 1.** Description of variables and parameters.

description^a^	variable/parameter	estimate (95% CI)
proportion of population with immune history *h* and carriers of class *i* serotypes	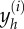	n.a.
proportion of population carriers of class *i* serotypes	*y*^(*i*)^	n.a.
proportion of population with immune history *h* and non-carriers	*x_h_*	n.a.
contact rate for low transmission serotypes among non-carriers	*β*_1_	0.085 (0.066,0.109)
contact rate for medium transmission serotypes among non-carriers	*β*_2_	0.090 (0.064,0.128)
contact rate for high transmission serotypes among non-carriers	*β*_3_	0.143 (0.071,0.289)
factor by which the rate of acquisition is reduced for carriers	*γ*	0.630 (0.407,0.975)
proportion that develop immunity after carriage	*p*	0.051 (0.032,0.082)
rate of carriage clearance for low transmission serotypes	*ν*_1_	0.049 (0.035,0.068)
rate of carriage clearance for medium transmission serotypes	*ν*_2_	0.039 (0.031,0.049)
rate of carriage clearance for high transmission serotypes	*ν*_3_	0.031 (0.025,0.038)
*per capita* rate of births and deaths	*μ*	0.00011^b^
vaccine efficacy	*f*	0.5^c^

^a^Rates *per capita* per day.

^b^Based on an estimated crude birth rate in The Gambia of 40 per 1000 per year.

^c^Estimated for PCV7 in [11].

**Table 2.** Loss and acquisition of serotypes in consecutive nasopharyngeal swabs. Nasopharyngeal swabs were taken every two weeks over 50 weeks, and serotypes are grouped as low, medium and high prevalence. The total number of transitions is reported, together with the observed probability of transition, and the probability predicted by the model (obs., obseved/pred., predicted).

from:	to: no carriage (obs./pred.)	low (obs./pred.)	medium (obs./pred.)	high (obs./pred.)	*N*^a^
no carriage		40 (0.037/0.038)	123 (0.114/0.114)	111 (0.103/0.100)	1075
low	50 (0.472/0.409)	9 (0.085/0.029)	12 (0.113/0.090)	15 (0.142/0.081)	106
medium	119 (0.317/0.354)	15 (0.040/0.029)	40 (0.107/0.087)	34 (0.091/0.085)	375
high	119 (0.285/0.304)	14 (0.034/0.029)	40 (0.096/0.095)	28 (0.067/0.087)	417

^a^Total number of pairs of consecutive swabs.

### 3.3. Alternative vaccines

**Figure 2.**
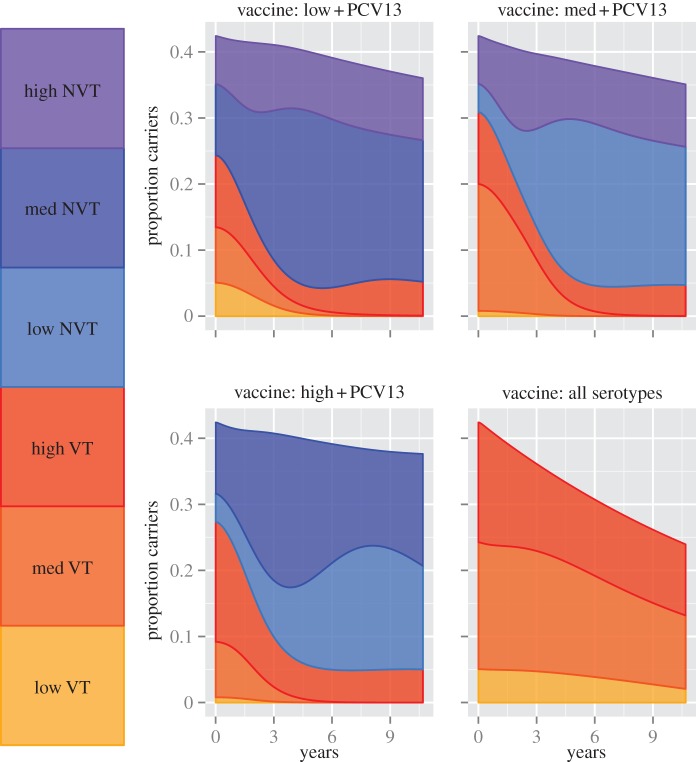
The prevalence of carriage after the introduction of four hypothetical vaccines. The vaccines protect against either low, medium or high prevlence serotypes as well as serotypes included in PCV13, or all serotypes. The vaccine efficacy against each serotype is assumed to be 50%, and coverage is 100%.

The model was used to explore the effect on pneumococcal carriage of four hypothetical vaccines that target different combinations of serotypes: (i) PCV13 and low prevalence serotypes; (ii) PCV13 and medium prevalence seroytpes; (iii) PCV13 and high prevalence seroytpes and (iv) all serotypes. We assumed 100% vaccine coverage and 50% vaccine efficacy against each serotype included in the vaccine. For vaccines 1–3, low and medium prevalence VT serotypes were eliminated, but there was serotype replacement, and therefore only a limited reduction in the prevalence of overall carriage. By definition, there can be no serotype replacement for vaccine 4, and there was consequently a significant reduction in carriage from 42.4 to 24.8%; however, low and medium prevalence serotypes were not eliminated by this vaccine (figure 2).

### 3.4. Sensitivity analyses

The model was fitted by grouping serotypes into three prevalence classes, assuming that all serotypes in a class have the same contact and clearance rate. To explore the effect of relaxing this assumption, we compared two six-serotype models. In the first model, all serotypes have different contact rates, whereas in the second model we grouped serotypes into low, medium and high prevalence classes, of two serotypes each, where the contact rate for both serotypes that share a class was the average of the contact rates used in the first model. We found that the dynamics of serotypes was similar in the two models (see electronic supplementary material, figure S1).

We used a model that allows for simultaneous carriage of two serotypes to investigate the effect of co-infection on serotype replacement (see appendix for a description of the model). The model was used to simulate pneumococcal carriage over a 2-year period (a shorter period was used because simulation of the co-infection model was computer intensive). For the simulation, we assumed full vaccine coverage and used the parameter values that were estimated for the superinfection model (table 1). We found that co-infection does not preclude serotype replacement (see electronic supplementary material, figure S2). In the first 2 years following vaccination, carriage of VT serotypes declined from 23.1 to 14.8%, and carriage of VT serotypes increased from 27.4 to 33.7%. The serotype replacement was slightly less than predicted under the superinfection model over the same period, where VT serotypes declined from 20.8 to 10.6%, and NVT serotypes increased from 22.3 to 30.9%.

## 4. Discussion

Projections from the model described in this paper suggest that PCV13 vaccination will lead to the elimination of low (1, 6A and 9V) and medium (4, 5, 7F, 14, 18C, 19A and 19F) prevalence VT serotypes and substantially reduce carriage of high prevalence serotypes (3, 6B and 23F) in The Gambia. However, the model predicts only a small reduction in the overall prevalence of carriage because the decrease in VT serotypes is counteracted by an increase in NVT serotypes. Initially, serotype replacement will be dominated by high prevalence NVT serotypes, but this will be followed by replacement with low prevalence serotypes as the population accrues immunity to the high prevalence NVT serotypes.

These predictions are broadly in agreement with studies that have compared the prevalence of carriage before and after the introduction of PCV vaccination, although there are as yet limited data on the impact of PCV13. Flasche *et al.* [12], for example, compared pre-vaccination data (2001/2002) with post-vaccination data (2008/2009) obtained from children under 5 years and their household contacts in a study conducted in the UK. The odds of VT carriage decreased by 93% post-vaccination, and serotypes 4 and 9V were not identified in any swabs (*n* = 382) after vaccination. NVT serotypes increased in this study so that there was no change in the overall level of carriage after vaccine introduction. Similar findings have been reported from pre- and post-vaccination carriage studies conducted elsewhere and are reviewed by Weinberger *et al.* [5]. Surveillance data are not yet available from The Gambia following the introduction of PCV13. However, Gambian children who had received PCV9 in the course of a randomized trial of this vaccine were more likely to carry NVT serotypes than control children and less likely to carry VT serotoypes [13].

Mixed results were obtained in a cluster randomized trial conducted in 21 Gambian villages. In this study, 11 villages were fully vaccinated with PCV7, and 10 control villages were partially vaccinated: only children 2–30 months and those born during the trial were vaccinated. The prevalence of VT serotypes was lower among vaccinated than control communities, but the prevalence of NVT serotypes was similar in the two arms of the trial [14,15]. In a comparison of prevalence at baseline and at six months post-vaccination, both VT and NVT serotypes decreased after vaccination, except among children aged 2–5 years where the prevalence of NVT serotypes increased.

In fitting the model to pre-vaccination data, we estimated that the probability of acquiring immunity after a single episode of carriage is 5% or, assuming immunity is acquired gradually, that the rate of acquisition of a serotype is reduced by 5% after each episode of carriage. The data are consistent with this finding since multiple episodes of carriage of the same serotype occurred frequently, particularly among children. For example, serotype 6B was carried on more than one occasion in 52% of children under 5 years, and some children carried serotype 6B up to six times during the year of follow up. This result is also supported by another Gambian study which found that only carriage of pneumococci of serotype 14 provided significant protection against subsequent reinfection with the same serotype, perhaps because this serotype induces a stronger immune response than other serotypes [16,17].

We also quantified the degree of competition between serotypes in the nasopharynx using the parameter *γ* in the model: for *γ* < 1, the serotype resident in the nasopharynx has a competitive advantage, while for *γ* > 1 the advantage goes to the establishing serotype. We estimated *γ* = 0.63, that is the rate of serotype acquisition is 37% lower among individuals that already carry a serotype compared with non-carriers. Auranen *et al.* [18] also found that the rate of acquisition was reduced for carriers compared with non-carriers, although the estimated reduction was much larger (91%) in this case. In a longitudinal study of Kenyan children, the reduction varied by serotype, but was generally less, with a maximum reduction of 52% observed for serotype 19F [10].

The relationship between carriage and disease is well established [19], and it is probable that the acquisition of *S. pneumoniae* in the naspharynx is a necessary step for progression to disease. Pneumococcal serotypes vary in their ability to cause disease [12,20], and PCVs target the most virulent serotypes. Surveillance data from countries that have introduced pneumococcal vaccination have shown that these vaccines are able to reduce the incidence of disease despite serotype replacement [4]. In The Gambia, serotypes targeted by PCV13 are associated with three quarters of the cases of pneumococcal disease [21], but their prevalence in the nasopharynx is comparatively low (e.g. 20% in our data). The vaccine is therefore also expected to reduce disease in The Gambia.

Surveillance for pneumococcal disease post PCV is currently underway in The Gambia, and further carriage studies will be conducted in due course. The accuracy of the model can only be fully assessed once these data become available. If the model can predict carriage post PCV in The Gambia, then the model could be used in other settings where the prevalence of carriage and composition of serotypes is different. Alternatively, the model might be used to investigate different vaccines, such as the protein vaccine that is currently being developed, which is expected to protect against all serotypes. We have explored scenarios involving alternative PCV vaccines, and our results suggest that carriage can be reduced by vaccines that provide partial protection against all serotypes. Interestingly, the model also predicts that low prevalence serotypes could persist in this situation. Intuitively this is because a vaccine that reduces overall carriage, will also reduce competition between serotypes in the nasopharynx that would otherwise drive low prevalence serotypes to extinction.

The model assumes that naturally acquired immunity is serotype-specific. The success of serotype-specific pneumococcal vaccines (polysaccharide and conjugate) supports the view that anticapsular antibodies can protect against invasive pneumococcal disease and carriage. The observation that anticapsular IgG is produced in response to pneumococcal carriage suggests that serotype-specific immunity can be acquired through carriage [22], and mathematical models of superinfection predict that serotype-specific immunity is required to allow coexistence of competing strains [8]. There is, therefore, substantial evidence in support of serotype-specific immunity. However, immunity may also be acquired in other ways. Possible mechanisms include antibodies or cellular response to non-capsular antigens. Lipsitch *et al.* [23] argue that the similar age distribution of invasive pneumococcal disease across serotypes is indicative of a non-capsule-specific immune response. The model presented here does not incorporate such an immune response. However, if a similar level of non-specific immunity can be induced by pneumococci of all serotypes, this should remain approximately constant over time in the population because vaccination has only a modest effect on the overall prevalence of carriage. It may therefore be unnecessary to model non-specific immunity in this setting.

Other models of pneumococcal carriage have been developed, and importantly, serotype replacement is predicted by all models alike. These models consider a limited number of serotypes [24] or model VT and NVT carriage without considering the dynamics of individual serotypes [25,26]. A recent simulation study by Cobey & Lipsitch [27] includes multiple serotypes, but this model was not fitted to data. By contrast, we propose a multi-serotype model that we fit to data and use to make predictions about the impact of PCV13 in the Gambian setting. In another recent study, Nurhonen *et al.* [28] fitted a micro-simulation model to carriage data from Finland. Their model is different in structure and is used in a different context, but they also predict that VT serotypes can be eliminated, and they predict a similar degree of serotype replacement.

The ecology of bacterial species within the nasopharynx is complex. Not only are there interactions between the 94 serotypes of *S. pneumoniae* but interactions may also exist between the different bacterial species that inhabit this ecosystem [29]. Mathematical models can potentially provide an important tool to help understand this complexity, particularly in the context of interventions, for example vaccination, that may act to disrupt existing equilibria.

## Appendix A

### A.1. Likelihood

An individual is in one of *n* + 1 states corresponding to no carriage or carriage of one of the *n* serotypes, we represent rates of transition between states by a matrix, *Q*, with elements *q_r_*_,*s*_ (*r*, *s* = 1,…,*n* + 1).

The transition matrix consists of component matrices *Q_i_*_,*j*_ with dimensions *n_i_* × *n_j_* (*i*, *j* = 0, 1, 2,…,*m*) that represent transitions from a state in prevalence class *i* to a state in class *j*, where we define the class *i* = 0 to be the non-carrier state so that *n*_0_ = 1.

We assume that the system is at equilibrium prior to the introduction of vaccination so that the rates of transition remain constant over time. The elements of *Q_i_*_,*j*_ are identical and given below, except where they are on the diagonal of *Q*, in which case they are defined as  so that the rows of *Q* sum to zero [30].

and

where  and  are respectively the equilibrium proportions of non-carriers and carriers of serotypes in class *i* and immune state *h*. The first rate represents serotype clearance and the remaining rates are for serotype acquisition. The rate at which a non-carrier acquires a particular serotype in class *j* is the product of two terms: the rate at which the individual comes into contact with class *j* carriers of the serotype , and the probability that that they are susceptible . Rates of acquisition for carriers are similarly defined, except that the rate is reduced by a factor *γ*.

Define *P*(*t*) as the matrix of transition probabilities associated with *Q* for an interval of length *t*. Using matrix notation, the forward equations imply that

Given *P*(0) = *I*, the identity matrix, the solution to this equation is

where *l*_1_,…,*l_n_*_+__1_ are distinct eigenvalues of *Q* and the columns of *B* are the associated eigenvectors (see [30] for further details).

Let *o_r_*_,*s*_ denote the observed number of transitions between *r* and *s* over the course of the study (50 weeks), and *p_r_*_,*s*_ the corresponding element of *P*, then assuming a Markov model the log likelihood is

### A.2. Model of co-infection

The original model does not allow for co-infection: it is assumed that a newly acquired serotype immediately replaces any existing serotype. Here, we extend the model to incorporate simultaneous carriage of two serotypes. In this model,  is the population proportion that carry a single serotype in class *i*, and  is the proportion that carry two serotypes from either the same *i* = *j* or different transmission classes *i ≠ j*.

and

We assume that co-carriers are equally likely to transmit either serotype; the force of infection due to class *i* serotypes among non-carriers in state *h* is then

The force of infection due to class *i* serotypes among carriers of a class *j* serotype in state *h* is

All other parameters are defined as in the original model.
